# A Rare Case of Lung Squamous Cell Carcinoma Metastasizing to an Anterior Abdominal Wall Schwannoma

**DOI:** 10.5146/tjpath.2025.14155

**Published:** 2026-09-15

**Authors:** Shahin Hameed, Shehla Basheer Kollathodi

**Affiliations:** Department of Pathology, Malabar Institute of Medical Sciences, Kerala, İndia

**Keywords:** Lung squamous cell carcinoma, Metastasis, Schwannoma, Abdominal wall lesion

## Abstract

Metastasis to benign tumors is an uncommon phenomenon, with schwannomas being rare recipients of metastatic disease. We report a rare case of a 60-year-old female with lung squamous cell carcinoma (SCC) developing metastasis to an anterior abdominal wall schwannoma. The patient was diagnosed with lung SCC and received three cycles of neoadjuvant chemoimmunotherapy. Follow-up Positron Emission Tomography/Computed Tomography (PET-CT) revealed a metabolically active primary lung lesion with metastatic lymphadenopathy and subcutaneous fat stranding in the left hypochondrial and lumbar region. She underwent thoracoscopy with pleural biopsies and excision of the anterior abdominal wall lesion. Histopathology confirmed metastatic SCC in the pleural deposits and a schwannoma in the anterior abdominal wall, harboring metastatic SCC foci confirmed by p40 immunohistochemistry (IHC). This case highlights the diagnostic challenges posed by metastasis to benign tumors and underscores the importance of thorough pathological evaluation.

## Objective

The objective of this case report was to describe a rare instance of tumor-to-tumor metastasis, where lung squamous cell carcinoma (SCC) metastasized to a benign schwannoma in the anterior abdominal wall. This report aims to highlight the rarity of metastasis to benign tumors, particularly schwannomas, and to raise awareness among pathologists and oncologists regarding this rare metastatic pattern to aid in accurate diagnosis and management.

## Case Report

A 60-year-old female, a non-smoker with no significant co-morbidities, presented with persistent cough and weight loss. A biopsy confirmed lung squamous cell carcinoma, and she was initiated on neoadjuvant chemoimmunotherapy (three cycles).

**Figure 1:**
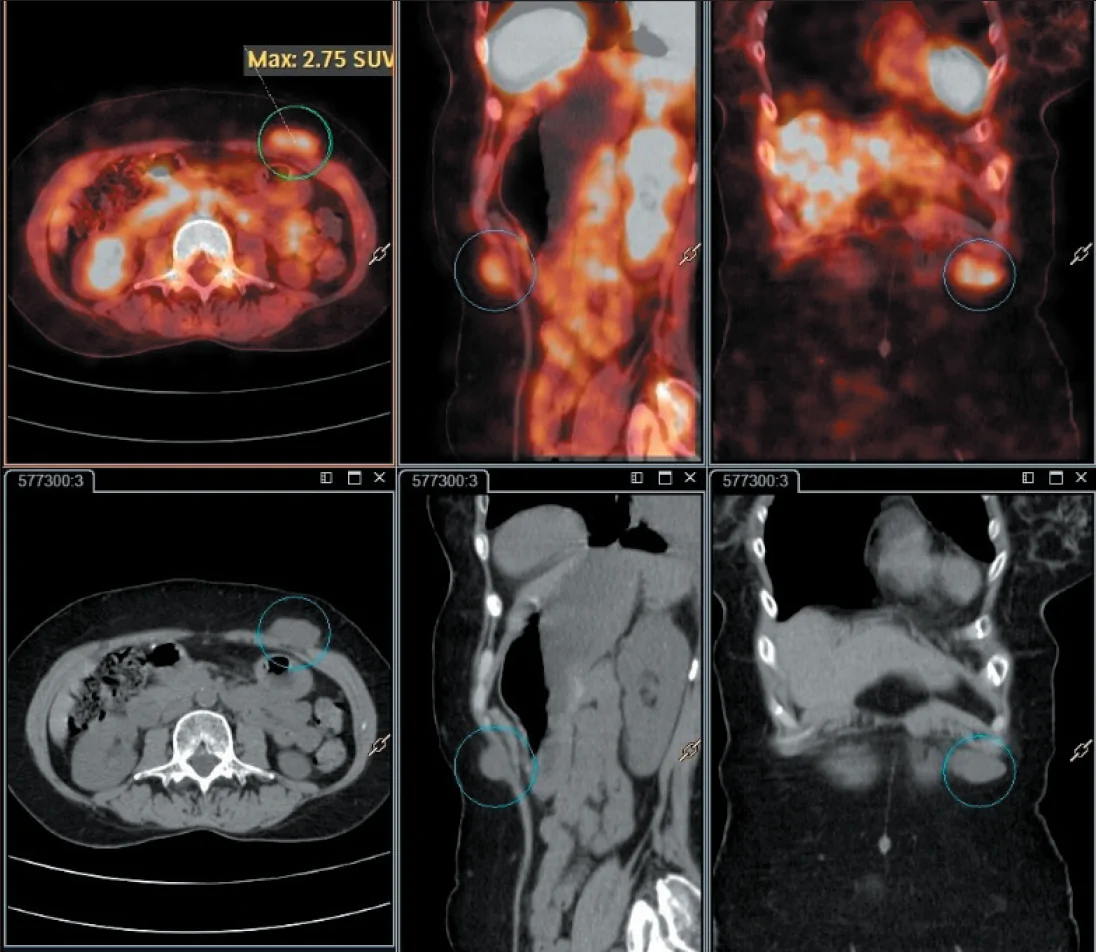
PET CT showing a metabolically active subcutaneous fat stranding was noted in the left hypochondrial region.

A follow-up PET-CT scan revealed a metabolically active soft tissue density lesion (3.3 × 3.1 cm) in the left lower lobe of the lung, with metabolically active left hilar and subcarinal lymphadenopathy. Additionally, metabolically active subcutaneous fat stranding was noted in the left hypochondrial and lumbar regions, raising suspicion for an atypical metastatic site (Figure 1).

**Figure 2:**
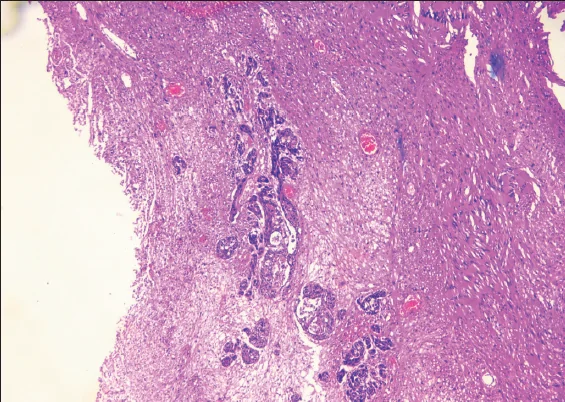
Schwannoma along with the small focus of metastatic squamous carcinoma (10X, H&E).

**Figure 3:**
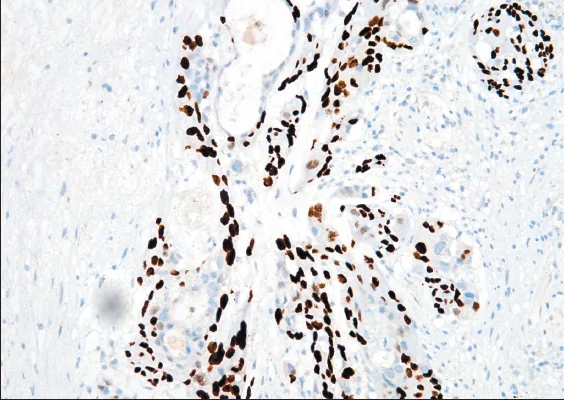
p40 positivity in the metastatic squamous cell carcinoma component (p40 antibody, 40X).

The patient underwent left thoracoscopy with multiple pleural biopsies and excision of the anterior abdominal wall lesion. Histopathology of the pleural nodules confirmed metastatic squamous cell carcinoma. The anterior abdominal wall lesion exhibited features of schwannoma with a small focus within the schwannoma that showed metastatic deposits of squamous cell carcinoma (Figure 2). Immunohistochemistry demonstrated diffuse and strong S100 positivity in the schwannoma component and p40 positivity in the metastatic component (Figure 3), confirming its origin from lung SCC. This case highlights the importance of histopathological and immunohistochemical evaluation in unusual metastatic presentations.

## Discussion

Tumor to tumor metastasis (TTM) is distinguished by a set of defined criteria proposed by Campbell et al. in 1968 (1), which include:

• At least two distinct neoplasms must exist

• True metastasis must be demonstrated histologically

• The metastatic tumor must grow within the host tumor rather than through contiguous spread

• The recipient tumor must be a benign or less aggressive neoplasm than the donor.

Schwannomas are benign peripheral nerve sheath tumors, and their involvement by metastases is extremely rare. Several mechanisms may explain why some tumors act as recipients for metastases that include high vascularity and low metabolic demand, and absence of immune surveillance, and some benign tumors may provide an immune-privileged niche that facilitates metastatic growth and expression of adhesion molecules like integrins and cadherins that promote selective homing to specific tissues (2).

Metastasis of lung cancer to benign tumors such as schwannomas is an exceedingly rare phenomenon. Schwannomas, benign tumors originating from Schwann cells of peripheral nerves, typically present in the head, neck, and extremities, and their involvement by metastatic disease is uncommon.

A literature review revealed sporadic cases, such as lung adenocarcinoma metastasizing to vestibular schwannomas (3). Notably, Slotty et al. reported a case of lung adenocarcinoma metastasizing to a dorsal root ganglion, emphasizing neural structure involvement; highlighting the rare occurrence of lung cancer metastasizing to the neural structures (4).

Metastatic lesions to schwannomas or schwannoma-like presentations can mimic other pathologies on imaging studies, leading to potential misdiagnosis. Advanced imaging modalities, while useful, may not always distinguish between benign and malignant lesions in such contexts (5).

Accurate diagnosis necessitates thorough histopathological evaluation, often supplemented with immunohistochemical staining. For instance, in the presented case, the metastatic squamous cell carcinoma (SCC) within the schwannoma was confirmed by positive p40 immunohistochemistry, a marker indicative of squamous differentiation (6).

The coexistence of metastatic carcinoma within a benign tumor like a schwannoma poses unique treatment challenges. Surgical resection remains a primary approach, but the presence of metastatic disease may necessitate adjunct systemic therapies tailored to the primary malignancy (7).

## Conclusion

While rare, the possibility of lung squamous cell carcinoma metastasizing to benign tumors such as schwannomas should be considered, especially when atypical presentations arise. Comprehensive histopathological assessment is crucial for accurate diagnosis, guiding appropriate therapeutic strategies, and improving patient outcomes.

## Conflict of Interest

The authors have no conflict of interest.
